# Barriers and facilitators to the implementation of clinical practice guidelines: A cross-sectional survey among physicians in Estonia

**DOI:** 10.1186/1472-6963-12-455

**Published:** 2012-12-13

**Authors:** Pille Taba, Marika Rosenthal, Jarno Habicht, Helvi Tarien, Mari Mathiesen, Suzanne Hill, Lisa Bero

**Affiliations:** 1 Department of Continuing Medical Education, University of Tartu, Puusepa 8, Tartu, 51014, Estonia; 2 WHO Country Office in Republic of Moldova, World Health Organization, 29 Sfatul Tarii Street, MD2029, Chisinau, Republic of Moldova; 3 Infectious Diseases and Drug Abuse Prevention Department, National Institute for Health Development, Hiiu 42, 11619, Tallinn, Estonia; 4 Estonian Health Insurance Fund, 10 Lembitu Street, 10114, Tallinn, Estonia; 5 Department of Essential Medicines and Pharmaceutical Policies, World Health Organization, 20 Avenue Appia, 1211, Geneva 27, Switzerland; 6 Department of Clinical Pharmacy, Institute for Health Policy Studies, University of California, San Francisco, 3333 California Street, Suite 420, San Francisco, CA, 94118, USA

**Keywords:** Clinical practice guidelines, Implementation, Estonia, World health organization, Barriers, Facilitators

## Abstract

**Background:**

In an era when an increasing amount of clinical information is available to health care professionals, the effective implementation of clinical practice guidelines requires the development of strategies to facilitate the use of these guidelines. The objective of this study was to assess attitudes towards clinical practice guidelines, as well as the barriers and facilitators to their use, among Estonian physicians. The study was conducted to inform the revision of the clinical practice guideline development process and can provide inspiration to other countries considering the increasing use of evidence-based medicine.

**Methods:**

We conducted an online survey of physicians to assess resource, system, and attitudinal barriers. We also asked a set of questions related to improving the use of clinical practice guidelines and collected free-text comments. We hypothesized that attitudes concerning guidelines may differ by gender, years of experience and practice setting. The study population consisted of physicians from the database of the Department of Continuing Medical Education of the University of Tartu. Differences between groups were analyzed using the Kruskal-Wallis non-parametric test.

**Results:**

41% (497/1212) of physicians in the database completed the questionnaire, comprising more than 10% of physicians in the country. Most respondents (79%) used treatment guidelines in their daily clinical practice. Lack of time was the barrier identified by the most physicians (42%), followed by lack of medical resources for implementation (32%). The majority of physicians disagreed with the statement that guidelines were not accessible (73%) or too complicated (70%). Physicians practicing in outpatient settings or for more than 25 years were the most likely to experience difficulties in guideline use. 95% of respondents agreed that an easy-to-find online database of guidelines would facilitate use.

**Conclusions:**

Use of updated evidence-based guidelines is a prerequisite for the high-quality management of diseases, and recognizing the factors that affect guideline compliance makes it possible to work towards improving guideline adherence in clinical practice. In our study, physicians with long-term clinical experience and doctors in outpatient settings perceived more barriers, which should be taken into account when planning strategies in improving the use of guidelines. Informed by the results of the survey, leading health authorities are making an effort to develop specially designed interventions to implement clinical practice guidelines, including an easily accessible online database.

## Background

Clinical practice guidelines are one of many tools that can be used to improve the quality of care provided by health professionals, especially when they are designed to support appropriate or necessary behavior change. Effective implementation of clinical practice guidelines requires a comprehensive approach beyond simply publishing and disseminating documents. An essential implementing first step is to assess local barriers in utilizing guidelines and develop strategies tailored to local circumstances [1].

Health care delivery in Estonia is provided by family doctors and their teams at primary care level and specialist services in outpatient and in-patient settings. The providers are contracted and services are purchased by the Estonian Health Insurance Fund, a mandatory social health insurance system covering approximately 95% of the population [2]. Numerous reforms have been introduced [2,3] and increased attention has been paid to quality of care, including usage of clinical guidelines [4]. The Estonian Health Insurance Fund (EHIF), in collaboration with other stakeholders, has launched a nationwide effort to further develop and implement evidence-based clinical practice guidelines. Until 2011, several institutions and health professional organizations in Estonia supported or developed national clinical practice guidelines. Although the first handbook for preparing guidelines was approved in 2004 [5], there has been variation in the format and quality of guidelines commissioned by EHIF. Over 90 guidelines in areas such as family medicine, cardiology, neurology, and oncology have been developed and are available on the public website for health care workers (*http://www.ravijuhend.ee/ravijuhendikasutajale/ravijuhendite-andmebaas/*), 40 of them prepared since 2003 following the first handbook for guideline preparation, steered by Estonian Health Insurance Fund, and additional 50 guidelines prepared using different approaches: initiatives by the medical associations, individual providers/specialties, interest groups and medical journals.

In order to register guidelines with the EHIF, they must contain an implementation plan. The EHIF facilitates implementation by developing outcome indicator measures that can be tracked through its IT systems, databases, and activities such as clinical audits. However, active implementation of guidelines has varied, depending to a large extent on the enthusiasm of the medical society concerned.

A 2007 review of studies of barriers to clinical practice guideline use found that the most frequently identified groupings of barriers were support/ resource barriers, cognitive/ behavioral barriers, health care professional/ physician barriers, system/ process barriers and attitudinal/ rational-emotive barriers. Less frequently identified barriers were related to the perceived quality of the clinical practice guidelines and evidence, or patient concerns [6]. Barrier assessments that have been conducted since the 2007 review identified fewer barriers, and the most frequent barriers were related to the guidelines themselves, patients, and support or resources [7-9]. For example, respondents were concerned that guidelines were not evidence-based, were not relevant to the population, were too complex, and they simply did not agree with the guideline recommendations. This finding emphasizes the importance of tailoring the guidelines to the local setting. Respondents were also concerned that guidelines would not meet the needs or characteristics of their patients. Lack of time and resources to implement guideline recommendations were additional major barriers. The identified barriers differed by type of guideline, demographics of providers, and type of practice setting. Interestingly, respondents in the more recent studies identified relatively few barriers related to physician characteristics other than a need for training and critical appraisal skills [7-9].

Most previous studies assessing barriers to guideline implementation have focused on disease-specific guidelines (e.g. hypertension or otitis media). Most were conducted among participants practicing in outpatient clinic settings. We did not identify any studies that were conducted in countries with limited human or financial resources. Therefore, the lack of studies applicable to the Estonian setting, where human resources are limited, highlighted the need to conduct an assessment of barriers to guideline implementation among Estonian physicians. The objective of this study was to assess attitudes towards clinical practice guidelines, barriers to and facilitators of guideline use among Estonian physicians in order to develop a tailored plan for guideline implementation at the country level. The survey was conducted as part of a larger project to review the Estonian guideline development handbook, processes, and roles of different stakeholders that was launched in 2010 by EHIF and WHO.

## Methods

Previous studies assessing barriers to guideline implementation have frequently used a survey methodology with responses to survey questions collected as dichotomous variables (e.g. agree/disagree) or 5-point Likert-type scales [6]. Recent barrier assessments have used a variety of theoretical frameworks to structure the survey questionnaire, with the Cabana 1999 framework mentioned most often [10]. Therefore, we developed an online survey assessing barriers in the following domains suggested by Cabana: resource/support barriers, system/process barriers and attitudinal/rational-emotive barriers of physicians and patients. We also asked a set of questions about suggestions to improve the use of clinical practice guidelines. These questions had five response options on a Likert scale ranging from ‘strongly agree’ to ‘strongly disagree’. The questionnaire also contained an area allowing respondents to write comments on their experiences with treatment guidelines in clinical practice. Two coders independently read all free text comments and grouped each comment into one of the following categories: resources, systems, attitudinal and other, and differences in opinions were resolved by concensus agreement between team members. The survey asked about treatment guidelines for a variety of conditions that are recognized and approved by the Estonian Health Insurance Fund (see *http://www.ravijuhend.ee/ravijuhendikasutajale/ravijuhendite-andmebaas/*).

The database of the Department of Continuing Medical Education of the University of Tartu was used to contact physicians who have attended educational courses. In Estonia, there are about 4600 actively practicing physicians, with a female preponderance among them, and about half of physicians attend regularly the educational courses of the Department of Continuing Medical Education. The survey was conducted during October and November 2010. The survey was sent twice by email and the online questionnaire, developed within the eformular system of the University of Tartu, was used to collect the data in table format. Following the first email, 324 respondents completed the questionnaire; following the second contact three weeks later, an additional 173 respondents completed it. A total of 497 physicians of the 1212 in the database completed the questionnaire, for a response rate of 41%. The sample includes more than 10% of all physicians in Estonia.

We hypothesized that attitudes about guidelines would differ by gender, years of work experience, and practice setting, as previous studies have found that barriers differ by physician characteristics [6-10]. The differences between groups were analyzed using the Kruskal-Wallis non-parametric test with a p value for significance of 0.05, followed by a post-hoc analysis with an adjusted p-value for significance of 0.008333 for comparison between single group pairs by years of work experience, workplace (hospital or outpatient clinic), and gender.

This study was evaluated by the Ethics Committee of the University of Tartu and classified as exempt from ethical review.

## Results

### Characteristics of participants

Of the 497 respondents, 285 (57%) worked only in hospitals (among them, 131 in the Tartu University Hospital), and 135 (27%) only in outpatient clinics as family doctors (106) or other specialists (29 doctors), and 77 (16%) in both types of settings. Among respondents, there was a preponderance of women (73% - 348/477), which is consistent with the demographics of Estonian physicians. 20% (101/495) had less than 10 years’ experience, 180 (36%) had 10-25 years’ experience, and 214 (43%) had more than 25 years’ experience. Most respondents used treatment guidelines in their daily clinical practice often (198/496, 40%) or sometimes (195/496, 39%). 20% of respondents (99/496) used guidelines seldom or never and only 4 (0.8%) were unaware of the treatment guidelines registered by the Health Insurance Fund.

### Attitudes towards treatment guidelines

As shown in Table 1, the majority of physicians surveyed agreed that the treatment guidelines recognized by the Estonian Health Insurance Fund are evidence-based, useful in their clinical practice, and a good tool for confirming diagnoses, initial treatment, and managing complicated cases. Approximately half of those surveyed agreed that the guidelines are convenient and easy to find. Hospital doctors agreed more favorably than those working in outpatient clinics that the guidelines are easily accessible (p = 0.0019). There were no differences in attitudes toward treatment guidelines by gender.

**Table 1:** Attitudes towards treatment guidelines

**Survey question(N** **=** **number of respondents)**	**Strongly agree**	**Somewhat agree**	**Neither agree nor disagree**	**Somewhat disagree**	**Strongly disagree**
Treatment guidelines are evidence-based (N = 494)	233 (47%)	206 (42%)	45 (9%)	10 (2%)	0 (0%)
Treatment guidelines are useful in daily clinical work and improve the quality of treatment (N = 497)	256 (52%)	210 (42%)	26 (5%)	5 (1%)	0 (0%)
Treatment guidelines include different aspects of a disease, and are a good tool for confirming diagnosis, starting initial treatment, and managing complications (N = 496)	212 (43%)	228 (46%)	40 (8%)	14 (3%)	2 (0.4%)
Treatment guidelines are convenient and the information is easy to find (N = 495)	130 (26%)	253 (51%)	51 (10%)	57 (12%)	4 (1%)

Physicians who have been in practice for the least amount of time had more favorable attitudes toward guidelines than more experienced physicians. Compared to doctors with more than 25 years’ experience, those with less than 10 years’ experience were more likely to rate guidelines as useful in daily practice (p = 0.004) and believe that the guidelines were evidence-based (p = 0.007).

### Barriers to use of treatment guidelines

The survey questions assessed barriers related to resources, systems, and attitudinal barriers of physicians and patients. As shown in Table 2, no specific barriers were seen as strong impediments to the use of clinical practice guidelines. Physicians were more or less evenly divided on whether resource issues were perceived as barriers to guideline use. The respondents were also divided on questions related to the availability of medical resources, patient resources, and time, although lack of time was the barrier identified by the most physicians (42%), followed by lack of medical resources for implementation (32%). The majority of physicians disagreed with the statement that guidelines were not accessible (73%) or too complicated (70%). The majority of physicians also failed to agree with a number of attitudinal barriers, including the belief that guidelines limit treatment options, flexibility in the way patients are treated, and physician autonomy. The physicians did not have strong opinions about patient attitudes toward treatment guidelines.

**Table 2:** Perceived barriers to guideline use

**Survey question(N** **=** **number of respondents)**	**Strongly agree**	**Somewhat agree**	**Neither agree nor disagree**	**Somewhat disagree**	**Strongly disagree**
**Resource barriers**					
Treatment guidelines are hard to implement in daily practice due to lack of medical resources (investigational abilities, etc.) (N = 496)	23 (5%)	137 (28%)	93 (19%)	205 (41%)	38 (8%)
Treatment guidelines are hard to implement in daily practice due to a lack of resources of patients (expensive medicines, etc.) (N = 494)	22 (4%)	130 (26%)	97 (20%)	214 (43%)	31 (6%)
There is no time to search for information (N = 490)	50 (10%)	157 (32%)	40 (8%)	172 (35%)	71 (14%)
**System barriers**					
Treatment guidelines are not accessible (N = 488)	7 (1%)	69 (14%)	55 (11%)	202 (41%)	155 (32%)
Treatment guidelines are too complicated and it is difficult to find the information (N = 489)	15 (3%)	74 (15%)	60 (12%)	256 (52%)	84 (17%)
**Attitudinal barriers**					
Treatment guidelines reduce doctors’ autonomy (a ‘cookbook’) (n = 492)	26 (5%)	94 (19%)	51 (10%)	229 (47%)	92 (19%)
Treatment guidelines limit treatment options (N = 483)	16 (3%)	44 (9%)	52 (11%)	267 (55%)	104 (22%)
Treatment guidelines limit flexibility and individual approach (N = 483)	29 (6%)	87 (18%)	52 (11%)	252 (52%)	63 (13%)
There is no need for treatment guidelines as treatment routines exist (N = 490)	5 (1%)	15 (3%)	32 (7%)	220 (45%)	218 (44%)
**Patient barriers**					
Patients do not want doctors to conform to treatment guidelines (N = 483)	4 (1%)	11 (2%)	179 (37%)	136 (28%)	153 (32%)

Compared to physicians practicing in hospitals, outpatient physicians, including family doctors, were more likely to experience a number of barriers, including a lack of time to search for information and lack of resources. Comparison of groups of family doctors and hospital based physicians is shown in Table 3. There were no differences between male and female physicians in the assessment of barriers to clinical guidelines. Compared to physicians with more than 25 years’ clinical experience, doctors with less than 10 years’ experience encountered fewer of the barriers assessed. Doctors with more than 25 years’ experience were more likely to feel that the guidelines were too complicated (p = 0.0006), limited their treatment options (p = 0.0002), flexibility and individual approach (p = 0.0001), and were more likely not to conform to guidelines (p = 0.00002) than physicians with less than 10 years’ experience. More experienced physicians were also more likely to have encountered barriers related to lack of medical (p = 0.0076) and patient (p = 0.0006) resources than those with less than 10 years’ experience.

**Table 3:** Comparison of barriers to use of treatment guidelines between family doctors and hospital based physicians

**Statement**	**Family doctors(n** **=** **106)**	**Hospital physicians(n** **=** **285)**	**p**	**Conclusion**
There is no time to search for information	2.40	3.39	p < 0.00001	For family doctors, lack of time to search for information is more serious barrier than for hospital physicians
Treatment guidelines are too complicated and it is difficult to find the necessary information	3.4	3.81	0.0003	Finding necessary information from guidelines is more difficult for family doctors
Treatment guidelines are hard to implement in daily practice due to lack of resources in medicine (investigation availabilities etc)	2.99	3.30	0.0117	Lack of resources in medicine is more serious barrier for family doctors compared to hospital physicians
Treatment guidelines are hard to implement in daily practice due to lack of resources of patients (expensive medication etc)	3.09	3.34	0.0340	Lack of resources of patients is the stronger barrier for family doctors

(Response options to the statements: 1 – strongly agree; 2 – somewhat agree; 3 – neither agree nor disagree; 4 – somewhat disagree; 5 – strongly disagree).

### Facilitators to use of treatment guidelines

There was strong agreement among 95% of respondents that an easy-to-find online database of guidelines would facilitate use (Table 4). Respondents also generally agreed that training courses on how to use guidelines, the availability of printed matter, provision of information through professional societies, and available consultancies (such as helplines) to answer questions about the guidelines would also facilitate the use of guidelines in practice. There were no differences in facilitators suggested by gender or work setting. However, physicians with less than 10 years’ experience were more likely to recommend an easy-to-find online database than physicians with more than 25 years’ experience (p = 0.0002).

**Table 4:** Perceived facilitators to guideline use

**Survey question(N** **=** **number of respondents)**	**Strongly agree**	**Somewhat agree**	**Neither agree nor disagree**	**Somewhat disagree**	**Strongly disagree**
An easy-to-find online database (N = 495)	349 (71%)	120 (24%)	14 (3%)	12 (2%)	0 (0%)
Special training courses (N = 496)	154 (31%)	203 (41%)	83 (17%)	54 (11%)	2 (0.4%)
Published materials (N = 492)	181 (37%)	200 (41%)	56 (12%)	49 (10%)	6 (1%)
Information through professional societies (N = 494)	216 (44%)	200 (40%)	40 (8%)	36 (7%)	2 (0.4%)
Available consultation to answer questions about the guidelines (N = 490)	191 (39%)	220 (45%)	60 (12%)	19 (4%)	0 (0%)

### Qualitative comments

21% (104/497) of respondents provided written comments. 74 respondents made 77 comments that could be grouped as attitudinal (n = 33), resource (n = 31), or system (n = 13) barriers. Two barriers mentioned that were not covered by the survey were concerns about malpractice liability and a lack of motivation to use guidelines. A number of additional themes emerged from the free-text comments. The respondents noted that guidelines for complex conditions, including guidelines for diagnosis, are particularly important. They felt that guidelines should be used consistently throughout Estonia. They also noted that guidelines would be useful in improving quality of care if they were regularly updated. The respondents likewise mentioned that clinical practice guidelines could be used to ensure that physicians were using an accepted standard of care. A few believed that the translation of internationally accepted clinical guidelines into Estonian would be acceptable for practice, but most respondents felt that international guidelines should be adapted to local conditions.

## Discussion

Assessing barriers and facilitators to the use of clinical practice guidelines is the first step in the local adaptation and uptake of evidence [11]. We surveyed Estonian physicians about barriers and facilitators to the use of clinical practice guidelines recognized by the Estonian Health Insurance Fund. We found that the majority of physicians were aware of and used the treatment guidelines. They believe the guidelines are evidence-based and are not concerned about guidelines that may limit professional autonomy (Table 1). The main reported barrier to guideline use was lack of time to identify guidelines. Lack of clinical and patient resources to implement the guidelines were also regarded as barriers (Tables 2,3). An easily located online database of clinical practice guidelines was suggested as the main solution to overcoming barriers to use (Table 4).

Family doctors that is the biggest group of specialists, were more likely to experience barriers related to a lack of resources, finding necessary information, or time to search for information (Table 3). There are a number of possible explanations for these differences. One might be related to the setting, with hospitals having better resources. Outpatient doctors might see greater number of patients, resulting in less time to search for information. The biggest difference in comparison of family doctors versus hospital based physicians, was demonstrated for the barrier related to lack of time to search for information. This confirms that limited time for one patient in outpatient clinic does not allow searching for information from treatment guidelines for making decisions.

Differences in education may affect an attitude to the use of guidelines. The medical specialists are graduated from one medical university in the country and the main differences could emerge by the graduating years. During the last decade, more emphasis is given to evidence based medicine within the curricula, but still limited.

Clinical practice guidelines have considerable potential to improve quality of health care as guidelines become integrated with information systems and electronic medical records [12]. A major barrier to such integration is the lack of computing infrastructure in many clinical settings. However, excellent integrated national information systems that are available to Estonian physicians suggest that clinical practice guidelines could be efficiently linked with electronic decision support systems in the country. Furthermore, Estonian physicians’ demand for an online database suggests that they are eager to take advantage of informatics solutions. However, physicians with fewer years of practice experience were more favorable towards clinical guidelines and online resources than those with more experience. As these physicians were likely younger and more comfortable using computer systems, it will be important to train older physicians to use these facilities with equal skills.

It is useful to use a well-established framework to assess local barriers and facilitators because they can vary extensively by setting and even change over time [13]. For example, barriers may be specific to the site of practice. In a survey of barriers to guideline use in hospitals, Simpson found that four hospitals reported the same doctor-related barrier as ‘most common’ and the remaining 10 hospitals reported three different doctor-related barriers, two nursing-related barriers and three organizational barriers as most common [14]. Even when barriers are consistent across sites, their influence may differ by type of health care professional [15] or by type of guideline, with each key recommendation having a unique pattern of barriers [6,8]. In our survey, doctors practicing in hospitals had easier access to guidelines and found them to be more understandable (Tables 2,3), identifying a need to distribute Estonian guidelines in ways that are equally suitable and accessible for outpatient clinicians. Although there were no differences by gender, more experienced physicians had more barriers to guideline use. Thus, tailoring an intervention to address the specific problems of access raised by outpatient and more experienced physicians could increase the use of guidelines in Estonia.

Resources, both human and financial, are needed for guideline development and implementation. Estonia has transitioned from a lower middle-income country in the 1990s to a higher middle-income country early in this century. However, the availability of clinicians and researchers who are trained in methods needed for evidence-based guideline development remains a resource constraint in Estonia. Few surveys of guideline implementation have been conducted in low-resource settings. However, a written survey conducted by Guindon and colleagues assessed how research evidence was used by practitioners in 10 low- and middle-income countries [16]. In general, the findings suggest that locally conducted and developed research played an important role, emphasizing the need for local capacity. A few questions were specific to clinical practice guidelines. In the Guindon’s study, only 12% of respondents (150/1249) had training in incorporating research evidence into a local guideline [16]. Thus, developing efficient mechanisms for guideline development that rely on adaptation of existing guidelines to the Estonian setting will promote the use of guidelines by involving local physicians in the process.

The major limitation of survey methods in assessing barriers to guideline use is that they are pre-specified by the investigators collecting the data. Therefore, we structured the questions in our barrier assessment according to a comprehensive theoretical framework [10] to help ensure that important factors were not excluded. Furthermore, our survey included open-ended questions to allow respondents to make additional comments. One limitation of our study was that the analyzed group was restricted to physicians who are included in the database of the Department of Continuing Medical Education as attended the educational courses. Thus, the study group represents more active part of physicians who are more open to new knowledge. Also, other health care professionals, particularly administrators and nurses, can play an important role in the successful implementation of guidelines. The response rate of the study was 41%, which is similar to other online surveys. In addition, our survey population consisted of more than 10% of the national sample of Estonian physicians.

## Conclusion

Estonian physicians feel that guidelines are a useful source of information and reported few barriers related to cognition, behavior, attitudes, systems, or processes. The main factors identified were related to the time needed to identify guidelines and the resources needed to implement them. A readily accessible online database of guidelines was viewed as a major facilitator; Estonia has the capacity to meet this need. In addition, guidelines must be easy to understand and relevant, particularly for older physicians in outpatient settings. The leading health authorities, in collaboration with a variety of stakeholders, are making an effort to develop a rigorous process for guideline development that will include the development of tailored interventions to implement the guidelines.

## Abbreviations

EHIF: Estonian Health Insurance Fund; WHO: World Health Organization.

## Competing interests

PT, MR, JH, HT, MM, SH and LB declare that they have no financial or non-financial interests that may be relevant to the submitted work.

JH is and SH was a staff member of the World Health Organization. JH and SH alone are responsible for the views expressed in this publication. They do not necessarily represent the decisions, policy or views of the World Health Organization.

## Authors’ contributions

PT designed the study, collected and analyzed data, and contributed to drafting the paper. MR collected and analyzed data, and contributed to drafting the paper. JH conceived the study, and contributed to drafting the paper. HT conceived the study, and contributed to drafting the paper. MM conceived the study, and contributed to drafting the paper. SH contributed to analyzing the data and drafting the paper. LB contributed to designing the study, analyzing the data, and drafting the paper. All authors read and approved the final manuscript.

## Pre-publication history

The pre-publication history for this paper can be accessed here:

http://www.biomedcentral.com/1472-6963/12/455/prepub
